# A Novel Framework for Data Assessment That Uses Edge Technology to Improve the Detection of Communicable Diseases

**DOI:** 10.3390/diagnostics14111148

**Published:** 2024-05-30

**Authors:** Mohd Anjum, Hong Min, Zubair Ahmed

**Affiliations:** 1 Department of Computer Engineering, Aligarh Muslim University, Aligarh 202002, India; mohdanjum@zhcet.ac.in; 2 School of Computing, Gachon University, Seongnam 13120, Republic of Korea; 3 Department of Zoology, College of Science, King Saud University, Riyadh 11451, Saudi Arabia

**Keywords:** infectious diseases, edge technology, data assessment scheme, disease detection, risk assessment, information analysis

## Abstract

Spreading quickly throughout populations, whether animal or human-borne, infectious illnesses provide serious risks and difficulties. Controlling their spread and averting disinformation requires effective risk assessment and epidemic identification. Technology-enabled data analysis on diseases allows for quick solutions to these problems. A Combinational Data Assessment Scheme intended to accelerate disease detection is presented in this paper. The suggested strategy avoids duplicate data replication by sharing data among edge devices. It uses indexed data gathering to improve early detection by using tree classifiers to discern between various kinds of information. Both data similarity and index measurements are considered throughout the data analysis stage to minimize assessment errors. Accurate risk detection and assessment based on information kind and sharing frequency are ensured by comparing non-linear accumulations with accurate shared edge data. The suggested system exhibits high accuracy, low mistakes, and decreased data repetition to improve overall effectiveness in illness detection and risk reduction.

## 1. Introduction

Communicable diseases are also known as transmissible diseases or infectious diseases. Some of the infectious diseases are COVID-19, Tuberculosis, AIDS, etc. Edge computing is widely used in identifying contagious diseases. An intelligent edge surveillance system uses edge computing to identify infectious diseases [1]. It is a remote sensing or monitoring system that is more effective and reliable than any other sensing system [2]. The smart edge system helps physicians, public health authorities, and hospitals to know the details about the affected person. This framework is mainly used to sense the communication chain of the infected people in society. This model detects the infected person and helps monitor their activities from the outside world. Edge computing stores the affected people’s data and records them safely and securely [3]. Infectious diseases or transmissible diseases can spread from one person to another by touching a contaminated surface or by physical contact with each other. The leading cause of infectious diseases is viruses and bacteria from animals or humans [4].

Communicable disease analysis is the main task performed in every healthcare department to provide a better environment for the people [5]. The analysis process helps to identify the affected or infected people from society by monitoring every person through a surveillance system. Without proper monitoring or analysis processes, infectious diseases will spread all around the surroundings and cause severe problems for the citizens of the whole world [6]. Fog computing is used to analyze contagious diseases. It is a real-time analysis process by the healthcare department with the help of collected records, which is used to provide a better environment for the people. It is more reliable and offers better performance when compared with any other analyzing process [7]. Edge computing is an information technology model that keeps the data storage and the computation process for the client closer. Edge computing is widely used to provide better service to the customer via networking technologies [8]. Diseases that are spread from one person to another by physical contact or touching contaminated surfaces are called communicable diseases or infectious diseases. Infectious diseases are more dangerous than non-communicable diseases [9].

To maximize efficiency as compared to conventional models and to improve estimation accuracy, deep learning models are frequently used to extract high-level spatial information [10]. They are also frequently used to identify and interpret biological data. The decision tree is a critical tool for examining predictions that may be used to efficiently and explicitly characterize beliefs. Despite its limits, it is a graph that shows every possible outcome using division techniques [11]. The COVID-19 pandemic presented unique challenges and opportunities to alter global healthcare systems. Under these circumstances, it is now necessary to use novel intelligence [12]. Technologies that offer the chance to provide virtual health services effectively. The theory and methods of edge computing, which help close the technological divide between network edges and the cloud, have emerged with the rapid rise of mobile communication. It can expedite the content delivery to raise the quality of networking service. It drives multimedia services across mobile networks with the help of system intelligence [13]. Artificial intelligence (AI) algorithms process and interpret large volumes of data, extracting insightful patterns and information that help with accurate diagnosis, therapy selection, and disease prognosis [14]. Healthcare practitioners can improve their decision-making processes and produce more individualized and successful interventions by utilizing AI-driven predictive modeling. Emerging technologies have transformed the field of infectious diseases, impacting different aspects of the ecosystem, such as diagnosis, monitoring, treatment of chronic illnesses, prevention, and tailored medicines [15]. The main contribution of the paper is stated below.

1. To present a Combinational Data Assessment Scheme (CDAS) to accelerate disease detection.
2. To improve early detection by using tree classifiers to discern between various kinds of information utilizing indexed data gathering.
3. To detect accurate risk and assessment based on information kind and sharing frequency; these are ensured by comparing non-linear accumulations with accurate shared edge data.
4. To improve overall effectiveness in illness detection and risk reduction by exhibiting high accuracy, low mistakes, and decreased data repetition.

The remaining part of the manuscript is divided into sections: Section 2 engages with related works, Section 3 covers proposed CDAS approaches and analysis, the performance analysis is covered in Section 4, and finally, Section 5 is covered with a conclusion along with future works.

## 2. Related Works

Dong et al. [16] proposed an edge perturbation method for predicting microRNA (miRNA)-disease association or the EPMDA method. It is used in the miRNA method for prediction. Structural Hamiltonian information is used to design a feature vector for each edge in the graph. The planned feature vector is used in the disease prediction process. Compared with the Human miRNA Disease Database, EPMDA is more effective and improves the value of AUC.

Wu et al. [17] proposed a learning framework for miRNA for the positive-unlabeled problem. For the negative extraction process, a semi-supervised K-means model is used. Training samples are generated using the sub-gagging method. The proposed method reduces the negative sample rate and helps find the exact names of the diseases using the positive sample set of information. The proposed method outperformed when comparing it with other traditional prediction methods, and the prediction accuracy rate was higher and more accurate than any other method.

M. Safa et al. [18] proposed a novel prediction method for cardio stress using a machine learning algorithm in IoT devices. The proposed method uses the K-nearest algorithm and supports vector machine approaches. Here, new information interacts with the old information to avoid the duplication of information that will be saved. The proposed framework outperformed the traditional prediction method by increasing the inaccuracy rate in the prediction process.

Pham et al. [19] proposed a new multiple-disease prediction method using a machine learning algorithm. This method helps to identify the relationship between the different types of diseases based on the categories. The proposed method helps analyze the graph by calculating both positive and negative sets and helps identify the symptoms of the disease. The experiment result shows that the proposed method outperformed the traditional method by increasing the multiple classification process and improving the efficiency rate of the prediction process.

Rahman et al. [20] found that to provide healthcare to all people, everywhere, technology is essential. To tackle the challenges of collecting, monitoring, and securely storing data on patients’ essential body parameters through sensor technology, a healthcare architecture based on blockchain is suggested. Elements such as an Ethereum-permissioned blockchain, an IoMT device, and a Markov state chain are utilized by the framework. The technology outperforms previous systems in terms of node and transaction scalability by an impressive 80%. The framework is evaluated in comparison to current methods for improved performance, and it employs smart contracts for access control.

Xu et al. [21] proposed a new pathogenic genes prediction method using a network embedding approach named multipath2vec. The pathogenic prediction process is most widely used for disease prediction in every medical healthcare center. A multipath method is used to identify the random walk in the gene–phenotype network. A learned vector is used to calculate the similarities of the unexpected path from the heterogeneous network—the proposed method, named the pathogenic genes prediction method (PGPM), results in high accuracy for the pathogenic prediction process.

Li et al. [22] invented a new prediction method named FCGCNMDA, which was a fully connected graph convolutional network for a mi-RNA disease-related approach. Edge weight is represented using a fully connected graph; then, it combines with mi-RNA features for disease prediction. AUC values are high when compared with traditional prediction models. The proposed FCGCNMDA method is more reliable and increases the exact miRNA disease prediction system.

A feature selection method was proposed by Khamparia et al. [23] and used a deep learning neural approach named genetic algorithm. Neuromuscular disorder prediction is performed using this method. The genetic algorithm identifies gene subsets, and the Bhattacharya coefficient method determines the most effective gene subsets. The proposed integrated method improves the accuracy rate and is more effective when compared with other integrated prediction methods.

Zhang et al. [24] proposed a new method for a miRNA–disease association named multiple meta-paths fusion graph embedding models. MiRNA–disease interactions are used to collect information about diseases. The graph embedding model calculates the info related to the miRNA disease. The proposed model is used as a self-learning approach for the disease prediction process. From the comparisons, it is seen that the proposed model outperformed the traditional prediction method.

Badidi, E [25] proposed Edge AI’s potential to enhance public health while reviewing its function in early health prediction. This article addressed the difficulties and constraints that Edge AI faces in predicting health outcomes early on. It also highlighted the need for further research to tackle these issues and how these technologies can be integrated into current healthcare systems to fully realize the potential of intelligent health technologies. It is also critical to keep up with new developments and moral dilemmas as Edge AI advances in early health prediction.

Al-Zinati et al. [26] introduced a redesigned bio-surveillance system that utilizes mobile edge computing and fog to detect how these technologies can be integrated into current healthcare systems to fully realize the potential of intelligent health technologies and localize biological threats. The order of fog nodes in the suggested architecture is responsible for compiling monitoring data from all across their respective regions and identifying any possible dangers. The evaluation results demonstrate the framework’s capacity to identify contaminated areas and pinpoint biological hazards. Furthermore, the outcomes demonstrate how well the reorganization mechanisms modify the environment structure to deal with the highly dynamic environment.

To solve the issues with manual blood smear examination in tracking patients and result verification, Kamal, L. and Raj, R. J. R. [27] suggested an improved convolutional neural network approach for automated blood cell recognition and categorization. The proposed method automatically detects whole blood cells in blood smear images by combining sophisticated image-processing methods and deep learning algorithms. With rigorous training and validation, the suggested model obtains remarkable metrics such as 91.88% accuracy, 91% precision, 91% recall, and an 88% F-score, outperforming traditional Computer-Aided Diagnosis systems in clinical labs.

Yadav et al. [28] provided a strategy for Computation Offloading using Reinforcement Learning (CORL) to reduce power consumption and latency in healthcare devices that use IoMT. By identifying the best resources to offload work to, the system overcomes the problems of low battery capacity and time restrictions caused by service delays. When tested in an iFogSim simulator with realistic assumptions, the experimental results demonstrate that the strategy reduces power consumption, delays data transmission, and makes the most efficient use of node resources in edge-enabled sensor networks.

Nandy et al. [29] introduced a novel healthcare system that utilizes Wearable Sensors (WSs) and an advanced Machine Learning (ML) model called Bag-of-Neural Network (BoNN) to remotely monitor health and anticipate the onset of diseases. Distributed edge devices gather patient health symptoms and preprocess data in the epidemic model. At centralized cloud servers, the BoNN model is used to detect COVID-19 disease on an improved dataset. On a benchmark dataset from Brazil called COVID-19, the system achieved a 99.8 percent accuracy rate.

Methods for edge perturbation, learning frameworks, multiple-disease prediction, healthcare architectures based on blockchain, methods for predicting pathogenic genes, and methods for feature selection are all covered in the research papers that are included in the text. Among the many healthcare-related topics covered in these articles are multipath2vec, disease prediction, and the prediction of pathogenic genes. New bio-surveillance systems that make use of mobile edge computing and fog are introduced, and edge AI shows promise for enhancing early health prediction. For automated blood cell recognition and categorization, an upgraded convolutional neural network method beats out the old Computer-Aided Diagnosis methods.

## 3. Proposed Combinational Data Assessment Scheme

**Figure 1:**
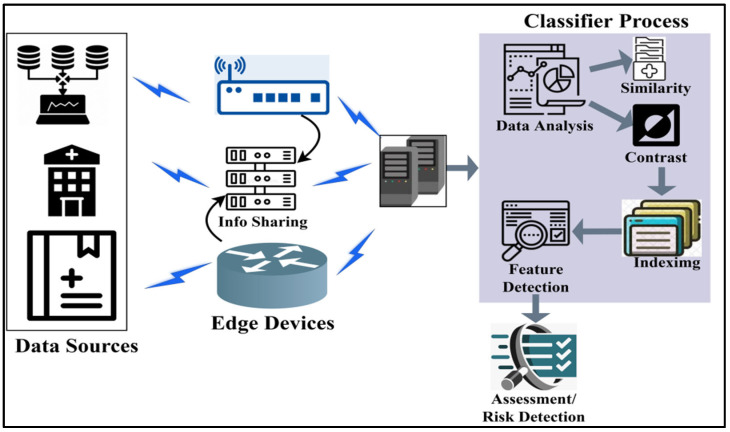
CDAS in Real-Time Environment using Different Classification Instances.

The proposed scheme relies on sharing data between the edge devices to prevent multi-source replications. Television, multimedia, graphics, cell phones, etc., do not transmit infectious diseases. Most cases of these infections spread through close personal contact with an infected person, contaminated objects, or respiratory droplets. In order to stop the dissemination of false information, it is essential to use reliable sources while discussing the spread of infectious illnesses. It helps extend and prevent false information about contagious diseases. A preventer is a group of software and hardware components that collect and process information accumulated from the healthcare center environment. The disease is controlled through various sources with sensor units to collect data such as frequency occurring, disease matching, data features, etc. Figure 1 portrays the proposed scheme in a real-time environment.

The deploying technology for analyzing disease-related information swiftly responds to the above problems. In the proposed CDAS, precise data sources and edge device control are prevented using the detection/recommendations of the analysis. The classifier performs similarity checks, difference data identification, and indexing in this analysis scheme. The indexed data are selected alone for feature extraction to identify the risks, as shown in Figure 1. The proposed CDAS improves disease detection swiftness, disease outbreak, risk assessment, and controlling infectious disease spread.

The edge disease consists of a specialized control unit that performs the functions of the edge devices (TV and MM) through edge devices and analysis (A). The functions of the edge devices are maintained using aggregators. The CDAS method serves as a data source and detection/recommendation. The aggregation unit rectifies the edge devices; therefore, it is predominant in controlling the spread and preventing false information from being built. It contains the spread of infectious diseases pursued using the data sources from the analysis (A). The CDAS analysis can be performed by four methods, namely, occurring frequency, classifier, data features, and matching. The input of data sources from the sensor (A) is functioned by the aggregation, then the matching function is transmitted. Therefore, CDAS is designed for actual data and replicating data analysis.

### 3.1. Data Analysis

The aggregators notice human and animal health conditions from infectious diseases. The input can be related to increased body temperature, coughing, fatigue, etc. In a noticing sequence, the data source received (*Ds*) derived as:

(1) $$Ds=A\frac{\pm (A_{max}\times A_{min})}{A}+A_{min}\;such\;that\;\varepsilon =\frac{1}{\sqrt{2\pi }A}\left[\frac{\left|\frac{A_{min}}{A_{max}}-\frac{\alpha }{A}\right|}{3\left|Ds-{Ds}^{*}\right|}\right]\}$$

where the variable α, denotes an active aggregator, and α∈A, $A_{max}$ and $A_{min}$ are the minimum and maximum data sources observed in varying instances. The variables $A_{max}$ keep information and previous information from being $A_{min}$. They are used to avoid noticing incorrect information, and prior information is used to prevent false information and previous information from being noticed. In the sense of a hoax, the wrong information is estimated as the number of mismatching analyses observed at continuous A observations. Therefore, some conditions of error Ds due to multi-source replications and disease detection swiftness α. This problem impacts the Ds at a given instance, for which the normalization is computed as:

(2) $$n\left(Ds\right)=\frac{{∆}^{*}}{{∆}^{*}{\left[\frac{A_{max}}{A_{min}}-\sigma_{s}\right]}^{2}}\;such\;that\;\sigma_{s}=\frac{2}{A}\sqrt{\frac{3}{A+j}\sum_{j+1}^{A}\left[{\left(\frac{Ds-{Ds}^{*}}{Ds}\right)}^{2}\times \frac{1}{\sqrt{2\pi }A}\right]}\}$$

The above equation specifies that the normalization comes after the maximum $A_{max}$ and standard deviation $A_{min}$ data sources observed standard deviation $\sigma_{s}$. Therefore, $n\left(Ds\right)$ is a normalized condition.

The symbols * and ∆ in Equations (1) and (2) stand to mean as follows: In mathematical equations, the symbol * usually means to multiply. The product of two integers, *A* and *B*, is represented by *A* * *B*. The delta sign, often used to indicate a change or difference between two values, is represented by ∆. The symbol ∆, when used in equations, can represent a change in a variable or a particular mathematical procedure tied to the idea of difference.

In contrast, it is the aggregation condition for which the proper estimation, therefore, Ds is normalized. In comparison, increment A by j as A+j is the aggregation condition for which the appropriate estimation action Ds is obtained. Based on Ds and $n\left(Ds\right)$, the instance of aggregation takes place, which is computed as follows:

(3) $$\varepsilon \left[Ds,n\left(Ds\right)\right]=\sqrt{{\left[\frac{n\left(Ds\right)}{Ds}\right]}_{a}^{3}-{\left[\frac{n\left(Ds\right)}{Ds}\right]}_{b}^{3}-\ldots -{\left[\left(j-\frac{{Ds}^{*}}{Ds}\right){∆}^{*}\right]}_{\alpha }^{3},\alpha \in A}$$

As per the above Equation (3), the instance of aggregation for a sequence until α is achieved in transmitting information from the healthcare centers the following example of aggregation is observed using machine learning. In an infectious disease scenario, data from the source must be transformed into controls to manage the spread of the disease effectively and ensure high accuracy for a prompt response. Additionally, it is essential to prevent the dissemination of false information (‘t’) to meet the healthcare requirements of edge devices. In this process, the early detection of infectious disease is allowed to protect humans and animals. In this way, the machine available used in infectious disease or $\varepsilon {\left[Ds,n\left(Ds\right)\right]}_{\alpha }$ is accessed. The output of the shared edge data of the machine learning is to find and separate the replicating data sequence through Ds evaluating and an operator−based analysis. Operator-based analysis refers to a method of data analysis that involves the use of mathematical operators or functions to manipulate and process data. This approach typically involves performing operations such as addition, subtraction, multiplication, division, comparison, or other mathematical functions on the data to derive meaningful insights or results. The first method of this learning is the frequency occurrence of the Ds instance if ε is observed. The concentration on achieving $\left(j-\frac{{Ds}^{*}}{Ds}\right){∆}^{*}$ at any instance is the output for separating the data. As per the process, two sequences of sample inputs of Ds at any varying instances of ϑ and τ are given as the input for the machine learning. Hence, in an ε aggregation, the sequence of disease detection takes place as per Equation (4).

(4) $$\begin{array}{l} \vartheta =Ds\tau =1\},the\;first\;instance\;is\;observed\;\vartheta =n\left(Ds\right)\tau \\ \;\;\;\;\;\;\;\;\;\;\;\;\;\;\;\;\;\;\;\;\;\;\;\;\;\;\;\;=\frac{\sigma_{s}}{{∆}^{*}}\},for\;the\;consecutive\;instances\;such\;that,\vartheta +\tau \\ \;\;\;\;\;\;\;\;\;\;\;\;\;\;\;\;\;\;\;\;\;\;\;\;\;\;\;\;=Ds,is\;the\;first\;data\;source\;where\;n\left(Ds\right)\\ \;\;\;\;\;\;\;\;\;\;\;\;\;\;\;\;\;\;\;\;\;\;\;\;\;\;\;\;\times \frac{\sigma_{s}}{{∆}^{*}},is\;the\;sequence\;of\;sample\;data\;sources\} \end{array}$$

**Figure 2:**
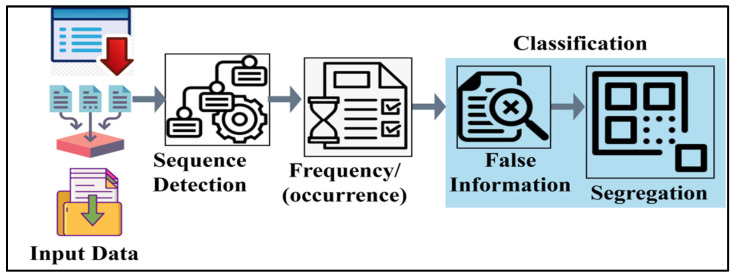
Data Analysis Process for Detection of False Data in Infectious Disease Monitoring.

The machine learning model assessment initiates from the sequence of sample inputs with the first edge device as Ds. This Ds is the ease of information analysis for evaluation; if the aggregation is observed in any varying instance, conjunction takes place. In Figure 2, the data analysis process is presented.

The conjunction process is performed using detected sequences based on occurrences. This occurrence factor is considered for identifying false (replicated) data. The specified data are segregated for further utilization. Here, the features associated with the classification are identified for detection (Figure 2). Therefore, in the machine learning used in infectious diseases, the shared edge data features are merged with a non-linear accumulation of data. The sequence of $\vartheta +\tau =n\left(Ds\right)\times \frac{\sigma_{s}}{{∆}^{*}}$ is analyzed to find the actual shared edge data in the edge devices. Machine learning classifies the process into two analyses of real data and replicating data based on the occurring frequency. The occurring frequency $\varepsilon {\left[Ds,n\left(Ds\right)\right]}_{\alpha }$ functions and its related things served by the edge device are discussed as per Equation (5).

(5) $$\begin{array}{ll} \varepsilon {\left[Ds,n\left(Ds\right)\right]}_{\alpha }= & \{{\left[\frac{n\left(Ds\right)}{Ds}\right]}_{a}^{3},\varepsilon {\left[Ds,n\left(Ds\right)\right]}_{j}>\varepsilon_{n\left(Ds\right)}{\left[\frac{n\left(Ds\right)}{Ds}\right]}_{b}^{3},\\ & \varepsilon {\left[n\left(Ds\right)\right]}_{j}\geq 0\varepsilon \left[Ds,n\left(Ds\right)\right]\\ & =X^{D}+\sum_{j+1}^{n}\left({\left[\frac{n\left(Ds\right)}{Ds}\right]}_{a}^{3}cos\;cos\frac{X^{D}\delta \left({\left[\frac{n\left(Ds\right)}{Ds}\right]}_{a}^{3}\right)}{\varepsilon_{n\left(Ds\right)}}+{\left[\frac{n\left(Ds\right)}{Ds}\right]}_{b}^{3}sin\;sin\frac{X^{D}\delta \left({\left[\frac{n\left(Ds\right)}{Ds}\right]}_{a}^{3}\right)}{\varepsilon_{n\left(Ds\right)}}\right)\varepsilon_{n\left(Ds\right)}\\ & =\frac{-X^{D}\pm \sqrt{\varepsilon \left[Ds,n\left(Ds\right)\right]+\alpha \left(j\right)}}{3cos\alpha_{j}}\} \end{array}$$

where the variable $X^{D}$ denotes the partial output of the edge device, and δ is the disease outbreak by the crowd observed in Ds. The frequency-varying instance can be analyzed by this occurring frequency method and then the classifier performs the next instance of functions. The classifier is used to identify the original data and replicate data in the edge device. If the classification is $\varepsilon {\left[n\left(Ds\right)\right]}_{j}$, then the method and its related thing are served by the machine learning. In this manner, the tree classifier method is deployed for classifying the data into two ways, namely, original data and replicating data, and then it is used for distinguishing contrast information analysis. For this purpose, two sequence data of Ds at any instance of ϑ and τ are used as the input for the machine learning. From a given instance, $\varepsilon \left[Ds,n\left(Ds\right)\right]$ followed by the tree classifier are analyzed by the machine learning method.

(6) $$R\left\{\varepsilon \left[Ds,n\left(Ds\right)\right]\right\}=-\vartheta (f)\pm \tau \left(Ds\right)-f\left(Ds\right)\pi \;such\;that\;\vartheta \frac{f\left(Ds\right)}{Ds}=\forall \left[Ds+\pi (f)\right]\tau \frac{Ds}{{∆}^{*}}=\forall \left[\vartheta -\pi (f)\right]\}$$

**Figure 3:**
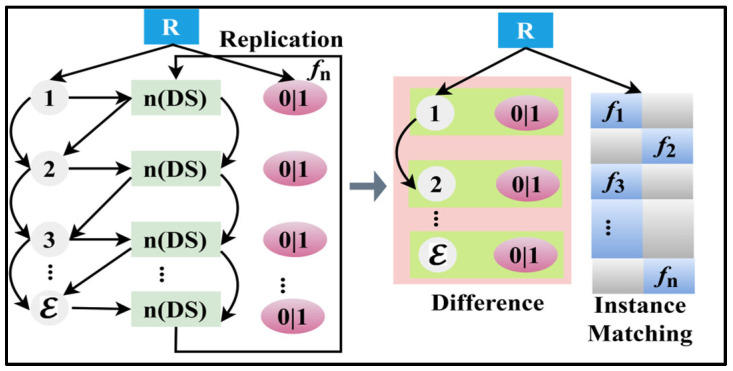
Error Analysis for Various Data Classification Process Sequences in Disease Detection.

As per the above Equation (6), the variable f is the output of the original data and π is the replicating data observed by the varying instance Ds. In the above equation, $\vartheta \frac{f\left(Ds\right)}{Ds}$ and $\tau \frac{Ds}{{∆}^{*}}$ are the related thing that is used for classifying the data of the $R\left\{\varepsilon \left[Ds,n\left(Ds\right)\right]\right\}$. In this manner, the aggregation method either satisfies $\vartheta \frac{f\left(Ds\right)}{Ds}$ or $\tau \frac{Ds}{{∆}^{*}}$ for all the sigmoid based $\left[Ds\pm \pi (f)\right]$ and $\left[\vartheta \pm \pi (f)\right]$. The true and false information of the above accumulation generates the non-linear ϑ±τ to achieve the above classification. Therefore, the aggregation process of $\vartheta \frac{f\left(Ds\right)}{Ds}$ and $\frac{Ds}{{∆}^{*}}$ and the non-linear accumulations of ${∆}^{*}$ and $n\left(Ds\right)$ together give the output of $R\left\{\varepsilon \left[Ds,n\left(Ds\right)\right]\right\}$ at its shared edge data. The machine learning of tree classifier analyzes ϑ,τ and $\varepsilon \left[Ds,n\left(Ds\right)\right]$, and it is followed by the sigmoid-based classification through ${Ds}^{*}$ and ${∆}^{*}$. Figure 3 presents the data classification process.

The replication factor is classified for the input data sequence based on n(Ds). Such classifications are performed for 0/1 augmentation in identifying the difference. This requires the matching of different instances. The occurrence $f_{1}$, $f_{2}$, …, $f_{n}$ are used for matching different instances. This is extracted from the replication classified as presented in Figure 3. The output of the original data using the swift response $\left\{Ds,{∆}^{*},\sigma_{s}\right\}$ is derived. Therefore, the first instance of replicating data provides indexed data collection and augments the early detection process. The replication processes are as computed in Equation (5) and (i.e.,) $\left[{(∆}^{*}=\sigma_{s})=1\right]$ is the output of the next instance, and hence, the occurring frequency is maintained without aggregation. Alternatively, the sequence of instance is observed, whereas the replicating data such as $\vartheta \frac{f\left(Ds\right)}{Ds}$ or $\tau \frac{Ds}{{∆}^{*}}$ impact the following data. Specifically, the occurring frequency of the above representation is either $\vartheta \frac{f\left(Ds\right)}{Ds}$ or $\tau \frac{Ds}{{∆}^{*}}$. The data sources of the inputs ϑ and τ are actual shared data such that the probability of matching the data is 1 or 2 for the sequence. Based on this example, the π conditions (i.e.,) $\pi >\frac{\sigma_{s}}{{∆}^{*}}$ or $\pi \leq \frac{\sigma_{s}}{{∆}^{*}}$ are analyzed. The π and its accumulations are matched for their features by preventing assessment errors and is computed as:

(7) $$\pi =2*\frac{\rho_{\tau }}{\rho_{\vartheta }}\;where\;\vartheta \frac{f\left(Ds\right)}{Ds}\;matching\;with\;Ds,if\;\pi >\frac{\sigma_{s}}{{∆}^{*}}\;else\;\vartheta \frac{f\left(Ds\right)}{Ds}\;matching\;to\;\sigma_{s}\;or\;{∆}^{*},if\;\pi \leq \frac{\sigma_{s}}{{∆}^{*}}\}$$

where $\rho_{\tau }$ and $\rho_{\vartheta }$ denotes the accumulations of ϑ and τ in the given equations. It is a way to identify if all the accumulated data matched for their features can be accumulated with both ϑ and τ. Now, the shared information between the edge device to overcome the multi-source replications for the output of $\pi >\frac{\sigma_{s}}{{∆}^{*}}$ and $\pi \leq \frac{\sigma_{s}}{{∆}^{*}}$ condition is derived in Equations (8) and (9).

(8) $$f_{1}={n\left(Ds\right)}_{1}f_{2}={n\left(Ds\right)}_{2}+{\left(\frac{\sigma_{s}}{{∆}^{*}}\right)}_{1}-{\left(\frac{\varepsilon }{\alpha }\right)}_{1}f_{3}={n\left(Ds\right)}_{3}+{\left(\frac{\sigma_{s}}{{∆}^{*}}\right)}_{2}-{\left(\frac{\varepsilon }{\alpha }\right)}_{2}\;such\;that\;f_{n}\;={n\left(Ds\right)}_{n}+{\left(\frac{\sigma_{s}}{{∆}^{*}}\right)}_{n+1}-{\left(\frac{\varepsilon }{\alpha }\right)}_{n+1},if\pi >\frac{\sigma_{s}}{{∆}^{*}}\}$$

(9) $$f_{1}={Ds}_{1}\pm {\tau \left(\frac{Ds}{{∆}^{*}}\right)}_{1}f_{2}={Ds}_{2}+{\tau \left(\frac{Ds}{{∆}^{*}}\right)}_{2}-{\left(\frac{\pi \times \varepsilon }{\alpha }\right)}_{1}f_{3}={Ds}_{3}+{\tau \left(\frac{Ds}{{∆}^{*}}\right)}_{3}-{\left(\frac{\pi \times \varepsilon }{\alpha }\right)}_{2}such\;that\;f_{n}\;={Ds}_{n}+{\tau \left(\frac{Ds}{{∆}^{*}}\right)}_{n}-{\left(\frac{\pi \times \varepsilon }{\alpha }\right)}_{n+1},if\pi \leq \frac{\sigma_{s}}{{∆}^{*}}\}$$

**Figure 4:**
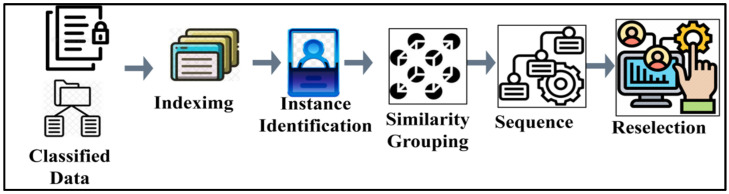
Data Feature Matching Process for Disease Detection.

The above-given representation is followed by the n sequence of the instance, where the early-detection Ds is augmented for classifying the output of the tree classifier. Therefore, the aggregation operation as in Equation (4) is analyzed for its frequency occurrence concerning the above-mentioned conditions of $\pi >\frac{\sigma_{s}}{{∆}^{*}}$ and $\pi \leq \frac{\sigma_{s}}{{∆}^{*}}$, utilizing the following determinations. These matching processes require some data features and also secure communicable disease information. Figure 4 presents the data feature matching process.

The classified data are indexed based on $f_{n},$ after which the similar data are grouped based on identified instances. The sequence is reselected if the similarity grouping fails and, hence, a new input is accessed for analysis (Figure 4). The data features in the sensitive information types and sharing frequency prevent similar data analysis, indexed data collection, and augmenting the early detection process. This requires a similar data analysis of f and π in Equation (6) for determining the instance of matching.

(10) $$\begin{array}{ll} R\left\{\varepsilon \left[Ds,n\left(Ds\right)\right]\right\} & =\left(Ds+\pi f\right)f-\left(\vartheta -\pi f\right)Ds-f\left(Ds\right)\pi ,\;for\;matching\;instance\\ & =\pm \left(Ds\right)f+\pi f^{n}-\vartheta \left(Ds\right)\{4A_{min}n\left(Ds\right)+\pi ,if\alpha =A\;and\;A_{max}=1\pm 4A_{max}n\left(Ds\right)+\pi \\ & =\frac{\rho_{\tau }}{\rho_{\vartheta }}+4A_{max}n\left(Ds\right)-2=\forall A_{max}n\left(Ds\right),if\frac{\rho_{\tau }}{\rho_{\vartheta }}=0\;and\;\alpha_{min}=1,and\;\alpha =A\} \end{array}$$

In this process, in the above Equation (10), $4A_{max}n\left(Ds\right)+\pi$ denotes the sequence of matching instances, and the assessment error indicates the end of the input data sources. Similarly, the next instance of matching for $R\left\{\varepsilon \left[Ds,n\left(Ds\right)\right]\right\}$ is designed for the similarity measures of the index, and the data are analyzed for the function $\pi \leq \frac{\sigma_{s}}{{∆}^{*}}$, as in Equation (11).

(11) $$\begin{array}{ll} R\left\{\varepsilon \left[Ds,n\left(Ds\right)\right]\right\} & =\pm \left(Ds\right)f+\pi f^{n}-\vartheta \left(Ds\right)=\pm Ds\left(Ds-\tau \right)+\pi (Ds-\tau {)}^{3}-\vartheta \left(Ds\right),\\ & Similarity\;measures\;of\;data=\pm {Ds}^{3}\left(2-\pi \right)+\left(Ds\right)\pi \left(3+2\pi \right)*\pi \tau^{3}-\vartheta \left(Ds\right)\\ & =\pm {Ds}^{3}\left(2-\pi \right)+\left(Ds\right)\pi \left(3+2\pi \right)*\pi \tau^{3}-\vartheta \left(Ds\right)\pm {Ds}^{3}+\left(Ds\right)\tau -\vartheta \left(Ds\right),\\ & if\;\pi \;is\;negligible\;\pi \to 1=Ds\left(\tau -\vartheta \right)+{Ds}^{3}=\left(Ds\right)\tau -{Ds}^{3}\left[\tau \left(\frac{Ds}{{∆}^{*}}\right)is\;assesment\;error\right]\} \end{array}$$

As per the above equation, the similarity measures the Ds analysis, as $\left(Ds\right)\tau -{Ds}^{3}$ is an assessment error during the sequence of $\pm 4A_{max}n\left(Ds\right)+\pi$. Therefore, the sequence of the instance as in Equation (11) occurs on $R\left\{\varepsilon \left[Ds,n\left(Ds\right)\right]\right\}$ as in Equation (10). Now, preventing false information and spreading control are initiated. This spread control represents the changes in the communicable disease of the edge device.

### 3.2. Spread Control

In the spread controlling process, the edge device takes the aggregation-based data analysis and decides the functioning part of the devices. The overall working of the device is synchronized based on $R\left\{\varepsilon \left[Ds,n\left(Ds\right)\right]\right\}$ outputs, respectively. Therefore, the initial spread control $X^{D}=1,$ such that if $X^{D}=2$, then the edge device functions through signaling from the aggregation. This depends on the π condition $R\left\{\varepsilon \left[Ds,n\left(Ds\right)\right]\right\}$ such that the probability of the spread control $\left(\rho_{X^{D}}\right)$ is computed as:

(12) $$\rho_{X^{D}}=\frac{{\left[count\left(X^{D}\right)\right]}^{\alpha }\times (\delta {)}^{n+1}*\left(Ds\right)f+\pi f^{n}}{\sum_{\alpha \forall A}{\left[count\left(X^{D}\right)\right]}^{\alpha }\times (\delta -\pi {)}^{n+1}}$$

From the given Equation (12), the probability of spread control is used to detect if the edge devices are working or not. If $X^{D}\geq 1\;\cup >\frac{\sigma_{s}}{{∆}^{*}}$, then the count of $X^{D}$ is incremented by one which means the edge device is working; otherwise, it is not working. Where δ represents the futuristic estimation of the replacement of $X^{D}$ between 1 and 2 and this method is computed as:

(13) $$\delta =\{\frac{\sum_{j+1}^{n}\pi_{j}}{i+\sum_{j+1}^{n}{\left[count\left(X^{D}\right)\right]}_{j}},if\;\pi_{j}<\rho_{X^{D}},j\in n\frac{2}{i+\sum_{j+1}^{n}{\left[count\left(X^{D}\right)\right]}_{j}},if\;\pi_{j}\geq \rho_{X^{D}},j\in n$$

This futuristic computation of communicable disease spread controlling following all the instances of n. From these appropriate detection/recommendations of δ outputs in an unsynchronized edge device control, the δ is derived from a sequential set of information instances. The communicable disease control output $\left(D_{z}\right)$ is computed as the non-linear matching of $\rho_{X^{D}}$, $R\left\{\varepsilon \left[Ds,n\left(Ds\right)\right]\right\}$ and π as:

(14) $$D_{z}=\{R\left\{\varepsilon \left[Ds,n\left(Ds\right)\right]\right\}\times \rho_{X^{D}}-\pi ,if\;\pi_{j}<\rho_{X^{D}},j\in nR\left\{\varepsilon \left[Ds,n\left(Ds\right)\right]\right\}\times \rho_{X^{D}}+\frac{\pi }{count\left(X^{D}\right)},if\;\pi_{j}\geq \rho_{X^{D}},j\in n$$

**Figure 5:**
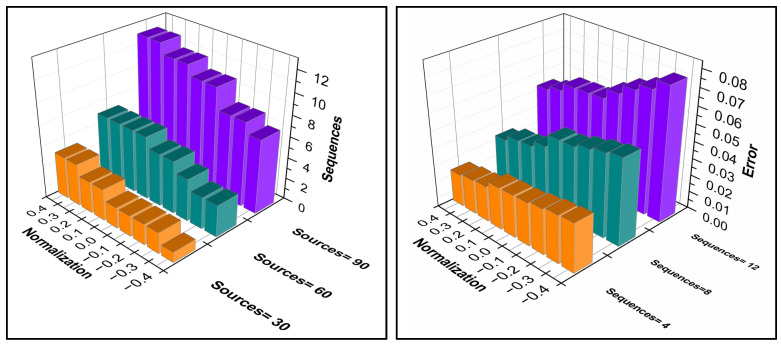
Identification of Overlapping Instances in the Sequences and Error for Different Normalization Factors.

In this process of detection, the result is represented as the state of the edge device of $\pi_{j}$ and $count\;\left(X^{D}\right)$ for all the n. The result of $D_{z}$ is based on $\pi_{j}<\rho_{X^{D}}$ and $\pi_{j}\geq \rho_{X^{D}}$. Hence, $\omega >\frac{\sigma }{∆}$ denotes a certain assessment of Ds in n. Similarly, the communicable disease spread controlling for retaining the high accuracy and also less replication occurs. In the Figure 5 series, the sequences and errors for different normalization factors are presented.

**Figure 6:**
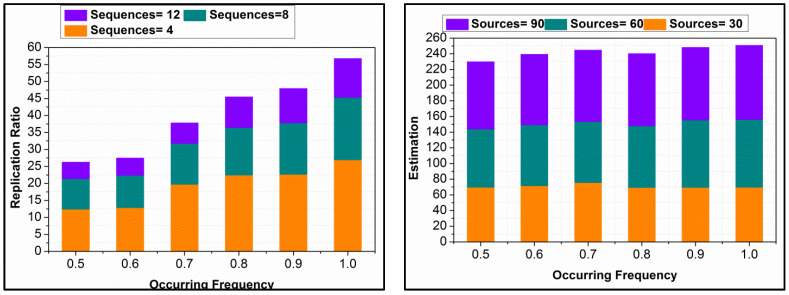
Analysis of ε[.] Maximization and Sequence Assigning for Reduced Replications.

An analysis of sequences and errors for different normalization factors is portrayed in Figure 5. The $n\left(DS\right)$ optimizes the detection sequences by mitigating ε. This is recommended based on the classification $R\left\{.\right\}$ This was pursued and hence the assessment errors were reduced. As the sequences migrate from −ve to +ve normalization, error reduces. However, the alternate matching for $f_{1}$ to $f_{n}$ addresses the errors and thereby the normalization is retained. The ε[.] induces further sequences in identifying and mitigating errors. Therefore, as normalization increases, the error is reduced, stabilizing the data analysis. Figure 6 presents the replication and estimation ratio for different occurring frequency values.

**Figure 7:**
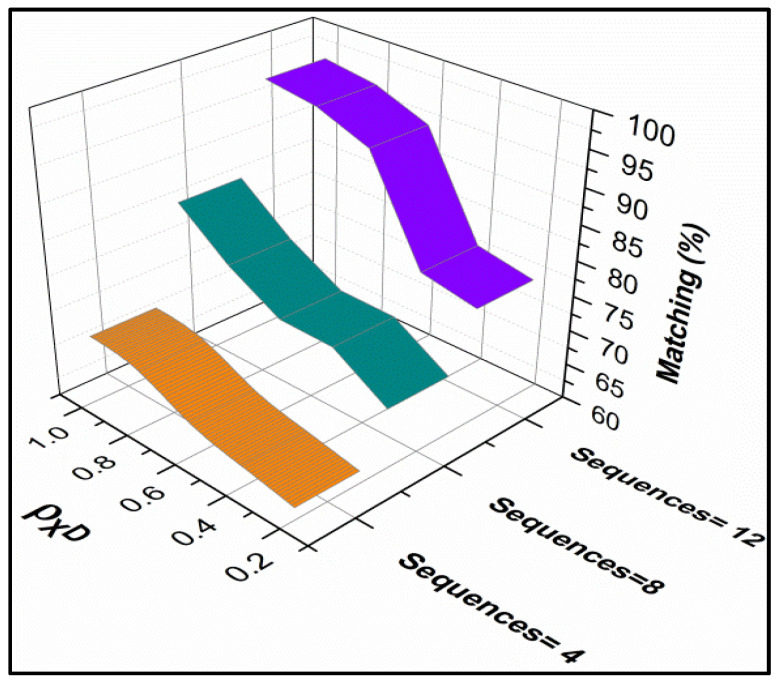
Matching Ratio Analysis for Different Spread Control Probabilities $\rho_{X^{D}}$.

The ε[.] in different DS inputs and R{.} functions reduce the replication by increasing the analysis. The estimations are based on Equation (7) followed by SD. This estimation increases the recommendations on classification for increasing the indexes and occurrences. The changes are updated in the subsequent classification instances, reducing errors. Therefore, the replications are confined without requiring additional computation. In the further estimations, $f_{1}$ to $f_{n}$ sequences are required to identify further ε[.]. This is required for reducing replications, through ε[.] maximization and sequence assigning. An analysis of the same is portrayed in Figure 6. In Figure 7, analysis for matching ratios for different $\rho_{X^{D}}$ is presented.

The matching ratio for different spread control probabilities is presented in Figure 7. As the classification instances vary the matching ratio increases for different ε[.]. This is due to the R{.} in the multiple iterations as classified by the learning process. The recurrent analysis is performed based on matching instances post the n(DS) based on ϵ[.]. This is however performed for −ve to +ve moves until the classification is before multiple iterations. Therefore, the matching increases as the $\rho_{X}^{D}$ is high regardless of the data sources.

For edge device infectious disease monitoring, Algorithm 1 coordinates spread control. Based on signs of disease transmission and device operation, it calculates the spread probability, $\rho_{X^{D}}$. Counts are increased if device usage or communication is above predetermined levels. The control decisions are guided by δ, a futuristic estimation. Considering $\rho_{X^{D}}$ and π, the spread of the disease control $D_{z}$ is calculated. To prevent duplication and stabilize data analysis, the program modifies device functioning and spread probability. Iteratively evaluating both illness incidence and gadget performance improves spread control tactics. Enhancing disease identification and response effectiveness in edge devices entails assessing spread probabilities, modifying device functionality, and reducing replication.
**Algorithm 1:** for Edge Device Spread Control in Infectious Disease Monitoring$\mathrm{Function}\;SpreadControl(Ds,n(Ds),\pi ,X^{D},\sigma_{s},{∆}^{*},\alpha ,A)$ $\mathrm{Input}:\;(Ds,n(Ds),\pi ,X^{D},\sigma_{s},{∆}^{*},\alpha ,A)$ $\mathrm{Output}:\;\mathrm{Probability}\;\mathrm{of}\;\mathrm{spread}\;\mathrm{control}\;(\rho_{X^{D}}$)). Futuristic estimation of spread control (δ). $\mathrm{Communicable}\;\mathrm{disease}\;\mathrm{control}\;\mathrm{output}\;(D_{z}$) Step 1: Calculate SpreadControl() $\mathrm{if}\;X^{D}>=1$$\;\mathrm{or}\;\pi >\sigma_{s}/{∆}^{*}$   $IncrementCount(X^{D})$     $\rho_{X^{D}}=CalculateSpreadControlProbability(X^{D},\alpha ,\delta ,\pi )$    $\;\mathrm{if}\;\pi <\rho_{X^{D}}$   $D_{z}=R\{\varepsilon [Ds,n(Ds)]\}\times \rho_{X^{D}}-\pi$    else:    $D_{z}=R\{\varepsilon [Ds,n(Ds)]\}\times \rho_{X^{D}}+\pi /Count(X^{D})$ $\mathrm{Return}\;D_{z}$ $\mathrm{Step}\;2:\;\mathrm{Function}\;CalculateSpreadControlProbability(X^{D},\alpha ,\delta ,\pi ):$     numerator=(Count(XD)^α)×(δ)^(n+1)×((Ds)f+πf^n)     denominator=Summation(α∀A)[Count(XD)^α×(δ−π)^(n+1)]     $\rho_{X^{D}}=numerator/denominator$      $\;\mathrm{Return}\;\rho_{X^{D}}$ $\mathrm{Step}\;3:\;\mathrm{Function}\;IncrementCount(X^{D})$     $\;\mathrm{Increment}\;\mathrm{the}\;\mathrm{count}\;\mathrm{of}\;X^{D}by1$ Step 4: Function Summation(α∀A)      Perform summation over all instances αforA $\mathrm{Step}\;5:\;\mathrm{Function}\;SpreadControlAnalysis(Ds,nDs,\pi ,X^{D},\sigma \_s,{∆}^{*},\alpha ,A)$      Computeδ based on Equation (13)      $\;\mathrm{Compute}\;D_{z}$ based on Equation (14)       $\;\mathrm{Return}\;D_{z}$
$\mathrm{Step}\;6:\;\mathrm{Function}\;Calculate\delta (\pi ,\alpha ,Count(X^{D}))$
     $\;\mathrm{if}\;\pi_{j}<\rho_{X^{D}},j\in n$
   $\delta =(\sum_{j+1}^{n}\pi_{j})/(i+\sum_{j+1}^{n}{\left[count(X^{D})\right]}_{j})$
       else         $\delta =\frac{2}{i+\sum_{j+1}^{n}{\left[count\left(X^{D}\right)\right]}_{j}}$
     Return δ

Data collection, pre-processing, analysis, and use are the four stages that make up the process flow for disease detection, followed by gathering data, cleaning them up, extracting features, training the model, evaluating it, discussing the results, and finally, training the model. Details regarding the dataset’s origins, infectious diseases, and data fields are provided. Addressing missing values, standardizing data, and eliminating duplicates are all part of data pre-processing. In order to detect diseases, feature extraction must be performed. During model training, algorithms, parameter adjustment, and validation procedures are utilized to train machine learning models. The study employs performance indicators such as sharing factor, replication ratio, error rate, and accuracy. The study of the results shows the results on improvement in sharing factors, correctness, decrease in errors, and replication. Disease detection and risk assessment are two areas where the suggested method shines, as discussed. See how the data assessment framework affects the efficacy of disease diagnosis and response with this step-by-step process flow.

Utilizing edge computing principles and devices, which are integral parts of our Combinational Data Assessment Scheme (CDAS), improves the efficacy of disease detection and response through the use of real-time data collection, rapid analysis, enhanced risk assessment, collaborative data sharing, and decentralized processing; it is clear that the suggested method is connected to edge technology.

## 4. Performance Analysis

The proposed scheme’s performance analysis is performed using the dataset [30] that contains information on different infectious diseases. The consistency in data availability with the observed and predicted values is used for similarity verification. This data source contains nine fields based on various categories. The experimental setup uses a standalone system that operates over eight data sources containing multiple instructions and 6–11 fields in common. The performance metrics used in this analysis are accuracy, error, replication ratio, and sharing factor. In the comparative analysis, the existing EPMDA [16], PGPM [21], FCGCNMDA [22], and miRNA [17] methods are used.

### 4.1. Accuracy

**Figure 8:**
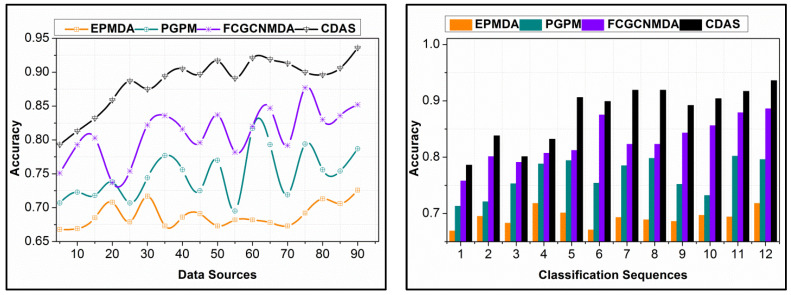
Accuracy Analysis of Varying Data Sources and Classification Sequences.

In Figure 8, the comparative analysis for accuracy under different data sources and classification sequences is analyzed. The proposed scheme identifies ε for the input Ds such that the process mitigates the replication through $R\left\{\varepsilon \left[Ds,n\left(Ds\right)\right]\right\}$. Therefore, the classification identifies non-replicated input sequences for improving data analysis accuracy. This process is aided until different conditional experiments are required. Contrarily, the index-based data analysis is performed under controlled futuristic estimation that requires less data for normalization. The further process is controlled by matching conditions defined in Equation (7) for which multiple information types are analyzed. This ensures error-less computations in proceedings with data analysis. The classification learning is pursued in different iterations satisfying the conditions in Equation (7). In this classification, α=A is verified throughout the n sequences in the $4A_{max}n\left(Ds\right)+\pi$ matching process. This reduces the errors in the intermediate classification sequences, for different input Ds. Therefore, the process improves the accuracy of obtaining matching based on similar data under defined parameters.

### 4.2. Error

**Figure 9:**
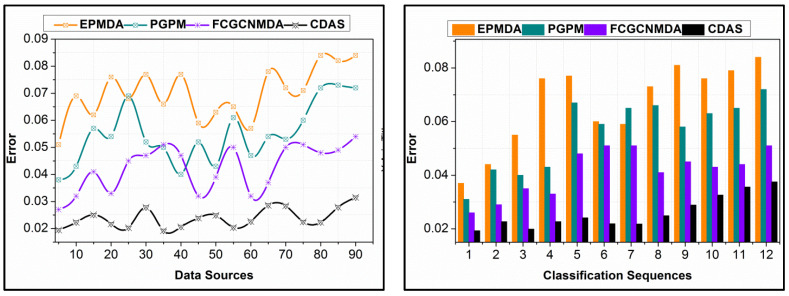
Error Analysis and Variation of Classification Sequences with Increasing Input.

The proposed scheme reduces errors in data analysis by segregating replication and non-replication instances over different classification sequences. The $\tau \frac{Ds}{{∆}^{*}}=\forall \left[\vartheta -\pi (f)\right]$ analysis for the classifier process is utilized for deviating errors in the continuous data. Contrarily, the changes in sequences require continuous classification to achieve high accuracy. The proposed sigmoid-based information classification refines the false data from the non-classified sequences, deviating errors. The intermediate $f_{1}$ to $f_{n}$ sequences verify the matching or un-matching sequences with distinct conditions in validating the accumulated data. Hence, the further Ds is analyzed using $\varepsilon_{n\left(Ds\right)}$ estimation, preventing replicated occurrence, and improving the accuracy. The error in non-replicating sequences is classified using occurring frequency, preventing $n\left(Ds\right)$. In this process, the previous occurrences and their classified sequence are identified for improving accuracy by reducing errors. The deviation $\sigma_{s}$ is mitigated by separating $\left(j-\frac{{Ds}^{*}}{Ds}\right){∆}^{*}$ such that the sequences are independently analyzed using machine learning. Therefore, as the input increases, the classification sequences are varied in confining the errors (refer to Figure 9).

### 4.3. Replication Ratio

**Figure 10:**
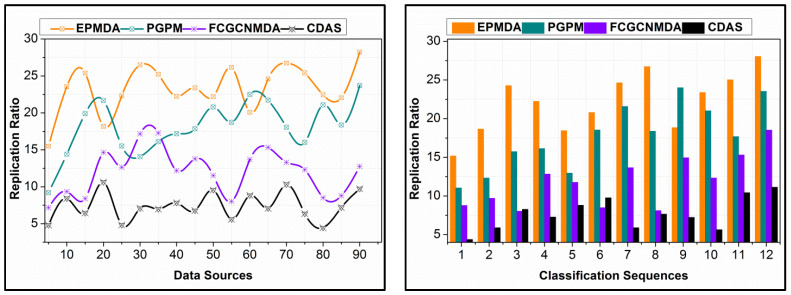
Replication Ratio Comparison for Different Data Sources and Classification Instances.

The proposed scheme’s replication ratio is comparatively less as presented in Figure 10 for different data sources and classification sequences. The proposed scheme identifies $\frac{\sigma_{s}}{{∆}^{*}}$ such that the overlappings in different instances are classified in the first analysis. This analysis is carried out for the partial edge device outputs, reducing errors. In this error analysis, first, the replications are mitigated using $\varepsilon \left[Ds,n\left(Ds\right)\right]$ predictions, $\forall \left[Ds+\pi (f)\right]$ and $\forall \left[\vartheta -\pi (f)\right]$. Further in the sigmoid classification, early detection of ${(∆}^{*}=\sigma_{s})=1$ is performed for identifying the false data. This identification is carried out using the learning process, in dividing multiple instances. Therefore, the validations in the replication are preceded using Ds or $\sigma_{s}$ matching. For the classified instances, the input from the data sources is validated based on the above matching conditions, as defined in Equation (8). After this process, $\pi >\frac{\sigma_{s}}{{∆}^{*}}$ and $\pi \leq \frac{\sigma_{s}}{{∆}^{*}}$ assessments are performed for identifying replicated sequences from multiple Ds. Therefore, the sequences are mitigated from different intervals and sequences, preventing false data. As the learning relies on the non-recurrent continuous instance, the replications are less in the proposed scheme as presented above.

### 4.4. Sharing Factor

**Figure 11:**
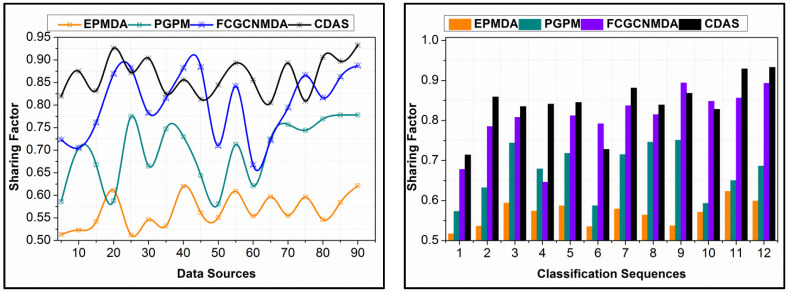
Data Sharing Factor from Various Data Sources and Classification Instances Analysis.

In Figure 11, the data-sharing factors from different Ds and classification instances are presented. The data-sharing factor in the proposed scheme is high compared to the other methods. The input data are analyzed for their falseness and replication before sharing; the analyzed data are shared based on $\rho_{X^{D}}$. This probability is used for identifying the data requiring and non-requiring control measures for improving the distribution. In this process, the $D_{z}$ results in conditional validation for actual data shared and required data for the control process. Therefore, the actual data requirements are upheld with the presence of false data, provided the distributed data is error-free. In this process, machine learning is completely utilized for futuristic data estimation, in determining the actual data requirement. The proposed scheme provides $R\left\{\varepsilon \left[Ds,n\left(Ds\right)\right]\right\}$-based data distribution, improving the sharing rate. For different Ds, the process is unanimous, preventing deviation-included data. Therefore, as the classification process increases, the sharing factor is leveraged compared to the other method, as represented above. In Table 1 and Table 2, the comparative analysis results are summarized.

The study follows a strict procedure to verify the accuracy, replication ratio, and sharing factor of the results. Determining the experimental setup, picking the right performance measurements, comparing the results to previous approaches, doing the math, drawing graphs, and talking about the results are all parts of this process. By checking that the results are credible and reliable, the author proves that the data assessment methodology proposed works to make illness diagnosis and response better. Graphs or tables are used to display the results visually so that they may be easily compared and understood. This procedure guarantees that the suggested data evaluation framework improves the efficacy of disease identification and response.

The Infectious Diseases dataset was used to evaluate the mathematical methods presented in the manuscript, which aim to forecast and prevent infectious diseases. Several diseases’ worth of data is included in the collection, which opens up possibilities for analysis and prediction modeling. In order to better understand the way, the suggested calculation methods detect disease patterns, reduce data replication, maximize data sharing, and minimize errors, the tests evaluate their accuracy, error rate, replication ratio, and sharing factor. It is essential to assess the strategies’ practicality in real-world situations.

The study developed a new Combinational Data Assessment Scheme (CDAS) using edge computing and AI to diagnose and prevent infectious diseases. Data collection, distribution, and analysis should be more precise and effective than traditional methods. Tree classifiers can improve indexed data-based early detection and discriminate data types. Considering data similarity and index measurements during analysis reduces assessment errors. Sharing frequency and information type can determine danger levels as compared to shared edge data. Minimal data replication, high precision, and low error rates improve efficacy. The authors submitted experimental results comparing CDAS to EPMDA, PGPM, and FCGCNMDA to support their claims. They found that CDAS increases data-sharing factors by 8.55%, reduces replication ratios by 11.83%, and increases accuracy by 14.77%. The study compares and quantifies the performance improvements of their new CDAS algorithm for infectious disease surveillance using edge computing resources. Future research could use this method with real-world edge deployments.

A real-world infectious disease dataset was used to test the CDAS approach. To understand the approach’s uniqueness and efficacy, more dataset information is needed. This includes data size, diversity and complexity, unique qualities or noise, and ground truth label and evaluation benchmark creation. This would show the complexity of real-world settings and the benefits of their edge computing and AI-based approach above previous methods. This would also inform CDAS expansion to other domains with similar data complexity.

### 4.5. Performance Metrics

**Figure 12:**
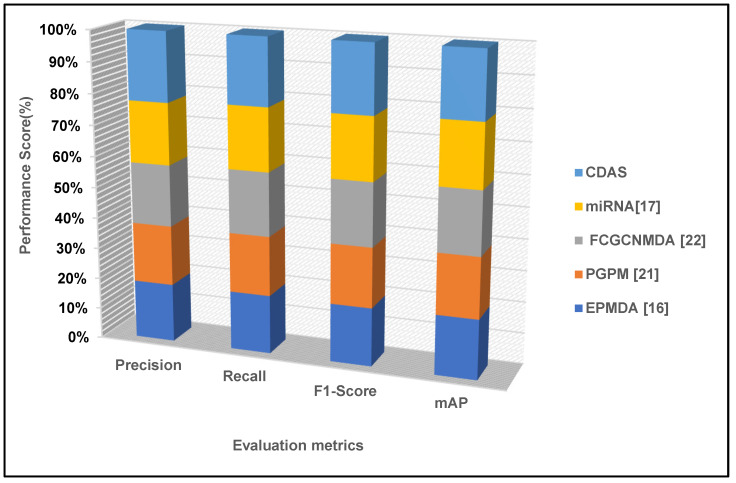
Comparison of Performance Metrics Across Various Prediction Algorithms.

Figure 12 shows a bar chart that compares various evaluation metrics across various algorithms and methodologies for a prediction or analysis job. Precision, recall, F1-Score, and mAP (mean Average Precision) are the evaluation measures displayed on the *x*-axis. Methods such as miRNAH7, CDAS, FCGCNMDA, PGPM, EPMDA, and PGPM are being compared. Generally speaking, CDAS performs the best across most metrics, as seen by having the highest bars, which represent the values for each statistic.

Findings: The proposed scheme achieves 14.77% high accuracy, 11.55% less error, 11.83% less replication ratio, and 8.55% less sharing factor.

Findings: The proposed CDAS improves accuracy and sharing factor by 13.6% and 10.35%, respectively. Moreover, it reduces the error and replication by 9.45% and 12.24% respectively.

In Table 3, the proposed method, CDAS is compared to edge devices in terms of resource constraints, computational intensity, data transmission requirements, latency considerations, and scalability and flexibility. CDAS must be optimized to efficiently utilize limited processing power, memory, and storage on edge devices, while edge devices typically have low-to-moderate computational intensity. CDAS data transmission requirements should consider bandwidth limitations and communication protocols of edge devices for seamless data exchange. Latency constraints should match the real-time processing capabilities of edge devices. CDAS should demonstrate adaptability to diverse edge computing environments and device configurations for optimal performance.

Several factors about computing resources and execution are compared in Table 3 between edge devices. The suggested Combinational Data Assessment Scheme (CDAS) approach is analyzed with features like resource constraints [25], computational intensity [22], data transmission [20], latency [28], along with scalability and flexibility [26].

(1) Resources Constraints:

In contrast with CDAS’s high resource requirements, edge devices often have minimal processing capability, memory, and storage. Following the basic principles of edge computing, the comparison implies that CDAS needs to be adjusted to make the most efficient use of the limited resources [25] on edge devices.

(2) Computational Intensity:

Compared to edge devices, having low-to-moderate computing capability [22], CDAS is defined as possessing moderate-to-high processing intensity. This comparison shows the significance of CDAS algorithms in being compatible with edge devices’ processing abilities in terms of complexity and real-time analytical capabilities for successful execution.

(3) Data Transmission:

The data transmission requirements of CDAS are minimal, in contrast to the limited bandwidth and protocol specificity of many edge devices discussed in [20]. The comparison shows that for CDAS and edge devices to share data seamlessly, CDAS data transmission needs should consider bandwidth constraints and communication protocols.

(4) Issues with Latency:

Contrasted with edge devices, CDAS is said to have latency [28] limitations ranging from low to moderate. According to the comparison, for decision making to be performed promptly, the latency restrictions of CDAS for detecting diseases should correspond to the real-time response rates that edge devices are capable of.

(5) Scalability and Flexibility:

Edge computing settings and configurations of devices can vary, but CDAS presents them as highly scalable and flexible [26]. Based on the comparison, it seems that CDAS needs to show that it can adapt to varied edge computing contexts and perform well with different configurations of devices to be deployed effectively in various situations. For edge devices, optimizing CDAS to meet their processing capabilities, data transmission needs, latency limits, and scalability considerations is crucial. To make the most of edge computing and get around any challenges edge devices may have, CDAS has to pay attention to these features.

## 5. Conclusions

To improve the efficiency of data distribution in the control of infectious illnesses, the paper presents a combinational data evaluation method. Using edge computing and AI methods, the suggested plan makes data collection, sharing, and analysis more efficient. To facilitate easy collection and analysis and avoid duplication and falsification, data sources are first identified. To verify the similarity measure among inputs and the available data and prevent data manipulation, a recurrent tree classifier learning technique is utilized. Indexing of non-replicated sequences comes next, after classification based on occurrence frequency. The likelihood that the indexed data will make it easier to share knowledge about controlling diseases is confirmed, and the process is then repeated for aggregated data sources until replication-free indexed data that are appropriate for sharing are generated. Based on experimental study, the suggested technique reduces error and replication by 9.45% and 12.24%, respectively, while improving accuracy and sharing factor by 13.6% and 10.35%, respectively, for various classification sequences.

## Figures and Tables

**Table 1:** Comparative Analysis Summary for Data Sources.

Metrics	EPMDA	PGPM	FCGCNMDA	CDAS
Accuracy	0.726	0.787	0.852	0.936
Error	0.084	0.072	0.054	0.0315
Replication Ratio	28.22	23.69	12.76	9.726
Sharing Factor	0.621	0.778	0.887	0.933

**Table 2:** Comparative Analysis Summary for Classification Sequences.

Metrics	EPMDA	PGPM	FCGCNMDA	CDAS
Accuracy	0.718	0.796	0.886	0.936
Error	0.084	0.072	0.051	0.0375
Replication Ratio	28.05	23.53	18.52	11.122
Sharing Factor	0.599	0.686	0.893	0.933

**Table 3:** Comparison of computing resources and implementation.

Consideration	Proposed Method (CDAS)	Edge Devices	Comparison
Resource Constraints	High	Limited	CDAS should be optimized to efficiently utilize limited processing power, memory, and storage on edge devices.
Computational Intensity	Moderate-to-High	Low-to-Moderate	CDAS algorithm complexity and real-time analysis capabilities should align with the processing capabilities of edge devices.
Data Transmission	Moderate	Limited	CDAS data transmission requirements should consider bandwidth limitations and communication protocols of edge devices for seamless data exchange.
Latency Considerations	Low-to-Moderate	Low	CDAS latency constraints for disease detection should be compatible with the response times achievable by edge devices for real-time decision making.
Scalability and Flexibility	High	Variable	CDAS should demonstrate adaptability to different edge computing environments and device configurations for robust performance across settings.

## Data Availability

The data that support the findings of this study are openly accessible at the following link: https://data.world/chhs/03e61434-7db8-4a53-a3e2-1d4d36d6848d, accessed on 20 March 2024.
